# Successful Treatment of a Catheter-Induced Superior Vena Cava Syndrome through Catheter-Directed Thrombolysis: A Case Report

**Published:** 2017-10

**Authors:** Reza Ghanavati, Ali Amiri, Nafiseh Ansarinejad, Shokoufeh Hajsadeghi, Hasan Riahi Beni, Seyyed Hashem Sezavar

**Affiliations:** 1 *Department of Cardiovascular Medicine, Rasoul-e-Akram Hospital, Iran University of Medical Sciences, Tehran, Iran.*; 2 *Shahid Beheshti University of Medical Sciences, Tehran, Iran.*; 3 *Department of Hematology and Medical Oncology, Rasoul-e-Akram Hospital, Iran University of Medical Sciences, Tehran, Iran.*

**Keywords:** *Vena cava, superior*, *Superior vena cava syndrome*, *Thrombosis*, *Thrombolysis*

## Abstract

Superior vena cava (SVC) syndrome is a medical condition resulting from the obstruction of the blood flow through the large central veins. Recently, central venous catheters have been reported as the increasingly common cause of this syndrome. We describe a 56-year-old woman with previous history of metastatic colon cancer, who had recently undergone central venous catheter insertion for her second chemotherapy course. Eight days following port insertion, she presented with signs and symptoms suggestive of acute SVC syndrome, which was successfully managed with catheter-directed thrombolysis. The pre-discharge transesophageal echocardiography and conventional angiography showed a patent SVC. The patient was discharged and remained asymptomatic over a 6-month follow-up. This case shows that catheter-directed thrombolysis may be used as a safe treatment for catheter-induced acute SVC syndrome in patients who have undergone catheter insertion in the central vein.

## Introduction

The superior vena cava (SVC) is a blood vessel with a thin wall and a low intravascular pressure and is surrounded by a constricted compartment. As there is no space for expansion, it can be compressed easily either by extraluminal lesions like tumors or intraluminal lesions such as thrombosis.^1^ SVC syndrome is a medical condition in which the blood flow of the head and the upper extremities is reduced due to stenosis or obstruction in the large central veins, including the SVC, subclavian veins, and brachiocephalic veins.^2^ Malignancy is the main cause of SVC syndrome, with the highest incidence related to bronchogenic carcinoma. During the past 2 decades, the increased use of central venous catheters has led to higher incidence rates (up to 40%) for nonmalignant etiologies of SVC syndrome. Regardless of the etiology, the most frequent presenting signs are neck and/or face swelling, upper extremity swelling, dyspnea at rest, and cough.^3^

Central venous catheter-related thrombosis may result in vascular and catheter obstruction as well as pulmonary embolism, formation of right-heart thromboembolism, and infection.^4^ Nowadays, central venous catheter-related thrombosis is a major problem in oncology practice. It may cause loss of the central venous access in 10% and pulmonary embolism in 10% to 15% of patients.^5^ At present, there is no published strategy vis-à-vis the prevention of thromboembolism and venous thrombosis in patients with central venous catheters.^6^

Benign SVC syndrome is mostly caused by thrombosis or by infection; it can be treated with catheter removal and anticoagulant therapy or antibiotics, respectively.^3^^, ^^7^ Several treatments such as placement of a metallic stent, percutaneous transluminal angioplasty, thrombolysis, venous grafting, and mechanical thrombectomy have been reported with varying degrees of success. Recently, catheter-based interventions have been drawn upon to remove the venous thrombus via minimally invasive strategies.^8^

Herein, we present a case of SVC syndrome following chemotherapy port insertion in a patient, who was successfully treated with catheter-directed thrombolysis (CDT). 

## Case Report

The patient was a 56-year-old woman with metastatic colon cancer of 4 years’ duration. Her past medical history was remarkable for diabetic mellitus and hypertension, both of which were under control. In June 2016, due to her increased CA 125 serum level, a second chemotherapy course, comprising oxaliplatin, leucovorin, bevacizumab, and 5-fluorouracil, was initiated after central venous catheter insertion (10 F silicone type) in the right subclavian vein. Four days after discharge, she started to present nausea and vomiting along with dyspnea at rest and chest pain. 

**Figure 1:**
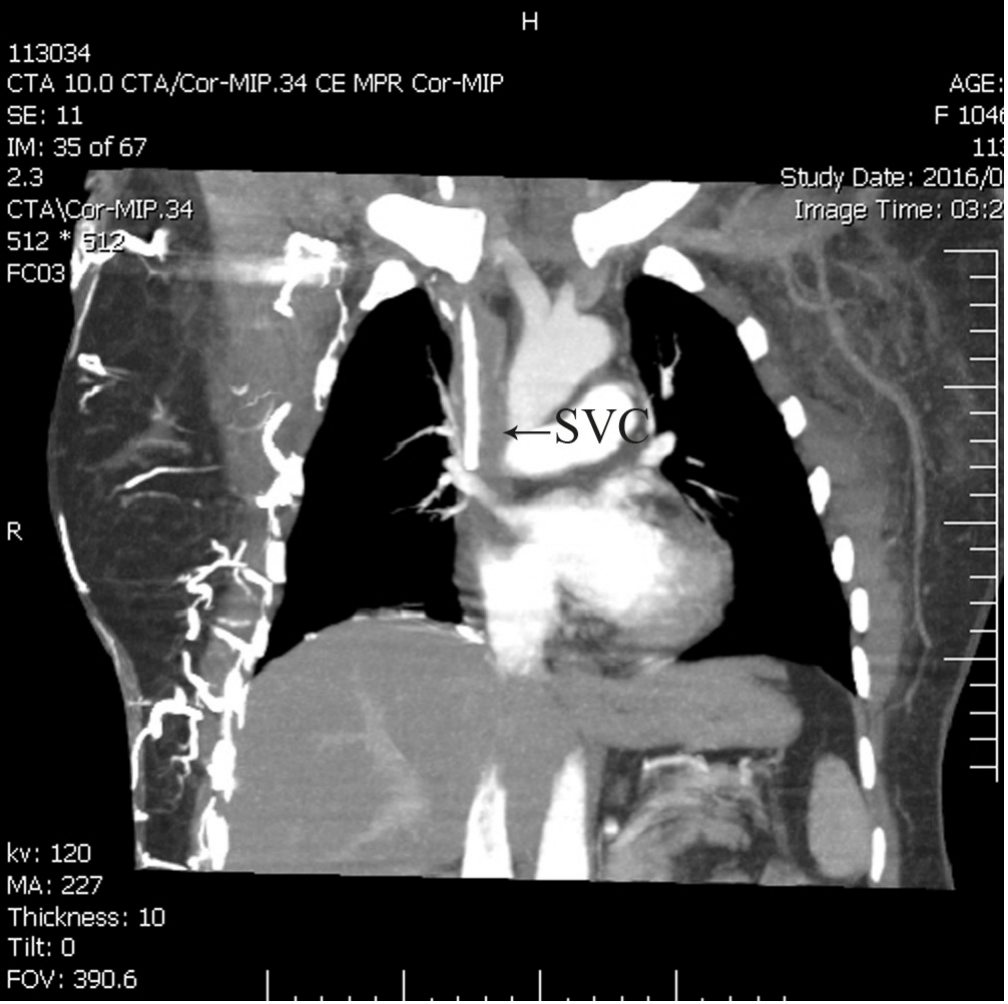
Computed tomography scan with contrast in the posterior anterior projection, prior to catheter-directed thrombolysis, showing an obstructed SVC (arrow) with many enlarged collaterals.

**Figure 2:**
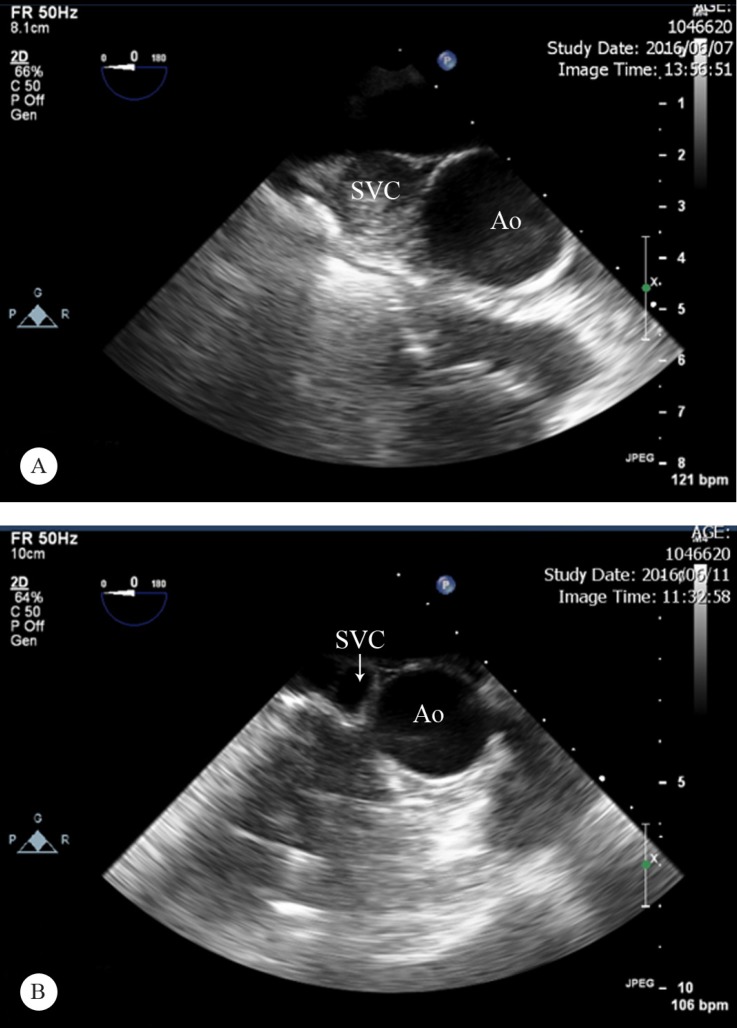
Transesophageal echocardiography in the upper esophageal view before catheter-directed thrombolysis (A), showing a dilated (20 mm) SVC totally obstructed by thrombus, and then a patent SVC (arrow) with complete resolution of thrombosis after treatment (B).

SVC, Superior vena cava; Ao, Aorta

**Figure 3:**
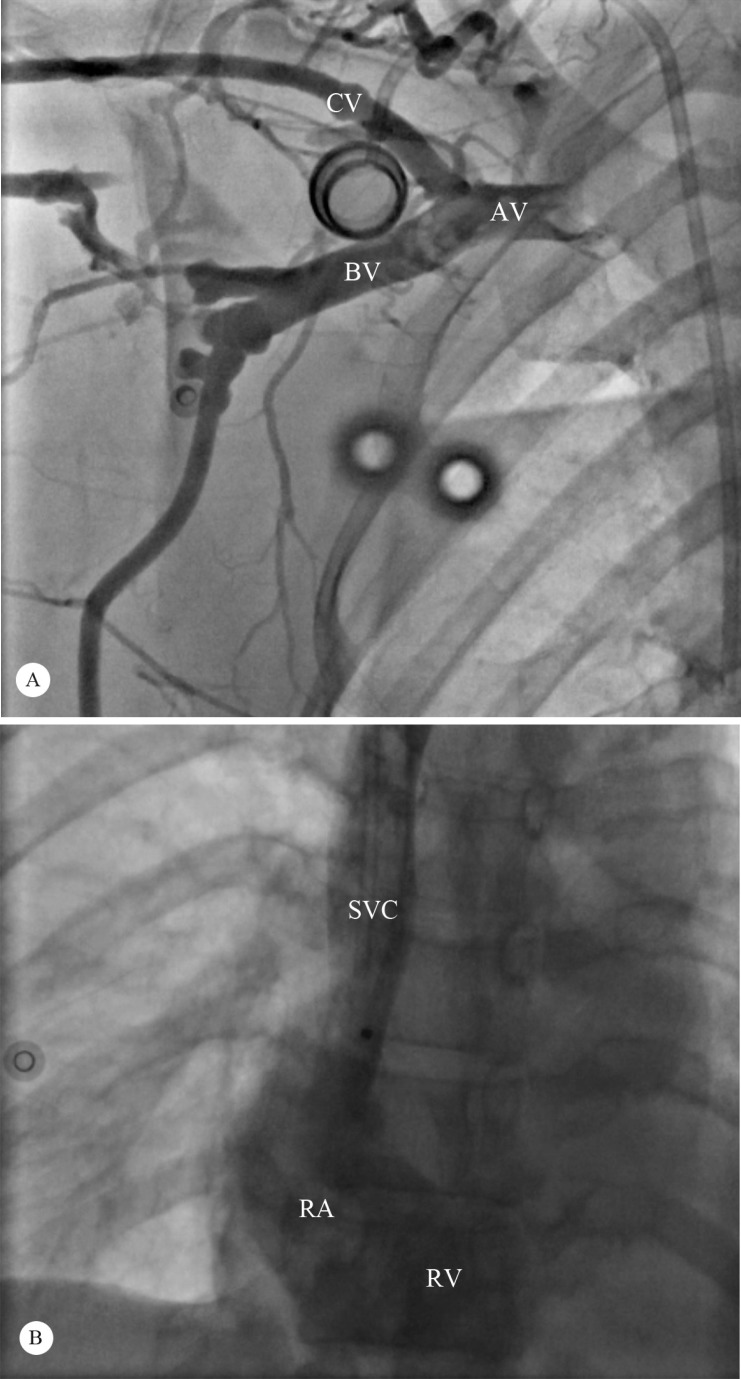
Coronary angiography in the left anterior oblique projection (A), showing absent filling of the SVC and the subclavian vein, consistent with thrombosis. After 20 hours of reteplase infusion, the follow-up coronary angiography in the anteroposterior projection (B) shows complete resolution of the thrombus.

AV, Axillary vein; BV, Basilic vein; CV, Cephalic vein; RA, Right atrium; RV, Right ventricle; SVC, Superior vena cava

Eight days later, the patient’s symptoms aggravated and she referred to the emergency department. On admission, the patient presented with 8 days’ history of shortness of breath and swelling of the face, neck, and right upper extremity, mostly in the morning time. On clinical examination, she had blood pressure of 122/76 mmHg, pulse rate of 78 beats/min, oral temperature of 36.5 ˚C, respiratory rate of 17 breaths/min, and oxygen saturation of 95% in room air. She was an oriented female, who appeared ill with a plethoric and edematous face, neck, upper chest, and right arm, accompanied with bluish lips. The lung auscultation was clear, and the heart sounds were normal. The chest X-ray and the coagulation profile were normal. A chest and neck computed tomography scan with contrast revealed an obstructed SVC with many enlarged collaterals and no sign of pulmonary embolism (Figure 1). Transthoracic echocardiography was performed and a large mass (1.5 cm × 1.1 cm) was seen in the right atrium, suggestive of vegetation or clot. Furthermore, transesophageal echocardiography was conducted, which demonstrated a dilated SVC (20 mm) totally obstructed by thrombus around the portal catheter with protrusion of the thrombus into the right atrium and mild pulmonary artery hypertension (pulmonary artery pressure = 40 mmHg) (Figure 2A). Accordingly, because of the patient’s negative blood culture and the absence of any evidence of catheter infection, the diagnosis of SVC thrombosis with extension to the right atrium was established. Subsequently, the patient was moved to the cath lab, where the coronary angiography showed absent filling of the SVC and the subclavian vein, consistent with thrombosis and an infusion catheter was placed adjacent to the thrombus via the left brachial approach (Figure 3A). Additionally, 0.5 IU/h of reteplase infusion was initiated over a 20-hour period. Finally, she was transferred to the intensive care unit for close monitoring. Over the next 20 hours, improvement in the patient’s clinical condition was notable. The post-CDT conventional angiography and transesophageal echocardiography illustrated complete lysis of the thrombus (pulmonary artery pressure = 36 mmHg) (Figure 2B and Figure 3B). After 7 days of hospital care, the patient was discharged asymptomatic, on warfarin treatment with an international normalized ratio (INR) between 2 and 3. She remained symptom-free over a 6-month follow-up. 

SVC, Superior vena cava

## Discussion

The prevalence of benign etiologies causing SVC syndrome is on the rise due to indwelling central venous catheters insofar as they allow the creation of a nidus for SVC thrombosis.^9^ Local endothelial damage, caused by the continuous movement of the catheter inside the vein or repeated central venous catheter insertion, leads to consequent inflammation, thrombin generation, hyperplasia of the intima, and fibrosis. Many factors can predict the risk of SVC syndrome secondary to the insertion of an indwelling catheter such as catheter location, catheter size, and indwelling time.^10^ In a study on 16 patients with SVC syndrome, Gray et al.^11^ reported that one of the factors capable of predicting the success of treatment was the initiation of treatment in a period of 5 days or less after the onset of symptoms.

Among central venous catheters, silicone rubber types (as was used in our patient) are associated with decreased thrombogenicity by comparison with polyvinyl chloride, polyethylene, and Teflon.^9^ The first-line treatment in patients with SVC syndrome due to intravascular devices is the removal of the device and systemic anticoagulation therapy in order to avoid thrombus propagation (as was carried out for the present patient). Mechanical thrombectomy or pharmacological thrombolysis can be used to decrease the risk of embolization and size of the obstruction when the patient has persistent symptoms despite systemic anticoagulation therapy or a huge thrombus burden.^7^

The optimal treatment for acute symptom relief in patients with SVC syndrome is endovascular stents. Thrombolysis or thrombectomy should be considered prior to stent development if it is dangerous or difficult to place a stent due to huge thrombi.^8^ Also, it is not recommended to place a stent for SVC syndrome created by benign diseases because of complications such as the possibility of stent migration, fracture or thrombosis, lack of long-term follow-up and longer life expectancy.^12^ Our patient showed no appropriate response to anticoagulant therapy, and the aggressive treatment was indicated because of her life-threatening symptoms.

CDT has reduced the need for systemic thrombolytic therapy as well as complications such as hematoma and gastrointestinal bleeding.^8^ Similar to our case, Dumantepe et al.^8^ and Cui et al.^7^ reported complete treatment of SVC syndrome following thrombosis using CDT. Also, Alkhouli et al.^13^ reported a case of inferior vena cava obstruction caused by thrombosis, which was successfully treated with CDT accompanied by angioplasty. In a study on 26 patients with SVC syndrome who received CDT (tissue plasminogen activator), Kee et al.^14^ reported that the response rate was only 15%. However, none of their cases was caused by catheters.

Grunwald et al.^15^ reported that in a comparison between alteplase, urokinase, and reteplase as a thrombolytic agent for CDT in the treatment of deep vein thrombosis, infusion time, success rate, and complications were not statistically significant; nevertheless, urokinase was significantly more expensive than the others. We did not perform transluminal angioplasty for our patient because her residual stenosis was less than 50% (not significant).^16^

## Conclusion

Catheter-directed thrombolysis can represent a potential therapeutic method in severe acute superior vena cava syndrome cases, especially in those with medical management failure or when systemic thrombolytic therapy or stent placement is too risky or contraindicated. In light of the present case, we believe that it seems reasonable to try catheter-directed thrombolysis in patients with catheter-induced acute superior vena cava syndrome.

## Notes:

***This paper should be cited as: ***Ghanavati R, Amiri A, Ansarinejad N, Hajsadeghi S, Riahi Beni H, Sezavar SH. Successful Treatment of a Catheter-Induced Superior Vena Cava Syndrome through Catheter-Directed Thrombolysis: A Case Report. J Teh Univ Heart Ctr 2017;12(4):188-191.
